# *Trypanosoma brucei* PUF9 Regulates mRNAs for Proteins Involved in Replicative Processes over the Cell Cycle

**DOI:** 10.1371/journal.ppat.1000565

**Published:** 2009-08-28

**Authors:** Stuart K. Archer, Van-Duc Luu, Rafael A. de Queiroz, Stefanie Brems, Christine Clayton

**Affiliations:** 1 Zentrum für Molekulare Biologie Heidelberg, DKFZ-ZMBH Allianz, Heidelberg, Germany; 2 Deutsches Krebsforschungszentrum, DKFZ-ZMBH Allianz, Heidelberg, Germany; Yale University, United States of America

## Abstract

Many genes that are required at specific points in the cell cycle exhibit cell cycle–dependent expression. In the early-diverging model eukaryote and important human pathogen *Trypanosoma brucei*, regulation of gene expression in the cell cycle and other processes is almost entirely post-transcriptional. Here, we show that the *T. brucei* RNA-binding protein PUF9 stabilizes certain transcripts during S-phase. Target transcripts of PUF9—*LIGKA*, *PNT1* and *PNT2*—were identified by affinity purification with TAP-tagged PUF9. RNAi against *PUF9* caused an accumulation of cells in G2/M phase and unexpectedly destabilized the PUF9 target mRNAs, despite the fact that most known Puf-domain proteins promote degradation of their target mRNAs. The levels of the PUF9-regulated transcripts were cell cycle dependent, peaking in mid- to late- S-phase, and this effect was abolished when *PUF9* was targeted by RNAi. The sequence UUGUACC was over-represented in the 3′ UTRs of PUF9 targets; a point mutation in this motif abolished PUF9-dependent stabilization of a reporter transcript carrying the *PNT1* 3′ UTR. LIGKA is involved in replication of the kinetoplast, and here we show that PNT1 is also kinetoplast-associated and its over-expression causes kinetoplast-related defects, while PNT2 is localized to the nucleus in G1 phase and redistributes to the mitotic spindle during mitosis. PUF9 targets may constitute a post-transcriptional regulon, encoding proteins involved in temporally coordinated replicative processes in early G2 phase.

## Author Summary

The unicellular protozoan *Trypanosoma brucei* is the causative agent of African sleeping sickness, responsible for over 100,000 deaths annually, and is related to other important pathogens (*e.g. Leishmania major* and *Trypanosoma cruzi*). Unusually, these organisms do not regulate their genes by changing the rate at which they are copied into RNA, but by changing the rate of RNA destruction or the rate of translation into protein. We identified an RNA-binding protein, PUF9, responsible for the accumulation of several RNA molecules at a specific time point in the cell division cycle, just after DNA replication. Correspondingly, the proteins encoded by these RNAs appear to function in the division of various cellular structures at this time point or shortly afterwards. Two of them facilitate replication of the kinetoplast (an organelle containing the mitochondrial DNA) while another was found in the mitotic spindle. Their temporal co-expression may stem from another unusual feature of trypanosomes: only one copy of the kinetoplast (and several other organelles) are present per cell, their replication being coordinated with cell division. Indeed, PUF9 may be important in the control of organelle copy-number because suppression of PUF9 resulted in cells with too many kinetoplasts, flagella, or nuclei.

## Introduction

The eukaryotic cell cycle is an ordered program of coordinated processes that mediate the replication of key cellular structures and their subsequent distribution to daughter cells. The proteins involved are often expressed at specific points during the cell cycle, for example dihydrofolate reductase (DHFR) [1] and histones [2], which are required before and after DNA synthesis, respectively, and whose regulation has been intensively studied. In most eukaryotes, such regulation might be expected to occur through transcriptional regulation. However, post-transcriptional regulation of mRNA, particularly in terms of differential message stability, is also important in ensuring that mRNAs do not remain for long after their usefulness has expired. For example, differential RNA stability of both DHFR [3],[4] and histone [5] mRNAs during the cell cycle plays an important part in the cell-cycle dependent expression of their protein products.

Post-transcriptional regulation is especially important for the kinetoplastids, an early diverging branch of model unicellular eukaryotes that includes many important human pathogens. This is due to an unusual feature of kinetoplastids: their mature mRNA transcripts are produced through cleavage and processing of large polycistronic RNAs, which are synthesized via unidirectional, RNA pol II transcription across large stretches of chromatin [6]. This unusual mechanism of mRNA generation precludes individual regulation of gene expression at the level of transcription. Instead, regulation of gene expression generally occurs via differential mRNA decay or other post-transcriptional mechanisms [7]. Despite this, kinetoplastids are capable of complex developmental regulation. For example, the model kinetoplastid parasite *Trypanosoma brucei* differentiates into two distinct cell types in the mammalian host and at least five in the Tsetse fly vector [8], and the different types have distinct gene expression profiles [9]. Kinetoplastids therefore constitute a class of eukaryotes that are able to proliferate, differentiate and adapt by means of entirely post-transcriptional gene regulatory networks, raising interesting questions about the nature of the pathways involved.

Several genes are known to be strongly differentially expressed during the kinetoplastid cell cycle. One group of co-regulated genes includes *DHFR*, topoisomerase 2 (*TOP2*) and replication protein-A (*RPA1*), whose transcript levels peak during S-phase due to the presence of a cycling element in the 5′ UTR with a consensus sequence (C/A)AUAGAA(G/A) [10]. This element can be functional in the 3′ UTR [11] or even in the pre-mRNA only [12], indicating that regulation can occur prior to mRNA maturation; and it also seems to be conserved in other kinetoplastids [13]. The *DHFR*, *TOP2* and *RPA1* transcripts, while originating from different parts of the genome, appear to belong to a post-transcriptional regulon [14] since they are co-regulated via common *cis*-regulatory motifs, and possess related biological functions.

Another interesting feature of the kinetoplastids is that organelles such as the mitochondrion, Golgi apparatus and flagellum exist as single-copy structures whose replicative cycles are tightly linked to that of the cell (reviewed in [15]). Hence, the expression of proteins involved in duplication of these organelles may also be regulated in concert with the cell cycle. This is illustrated by the replication of the kinetoplast, which is a distinctive structure housing the mitochondrial DNA (kDNA), a network of concatenated open-circular DNA minicircles and maxicircles and associated proteins. The replication of the kinetoplast is coordinated with the cell cycle and involves several dedicated kDNA-processing proteins, some of which are members of the post-transcriptional regulon mentioned above. Kinetoplast DNA ligase alpha (LIGKA) is involved in kDNA replication and is regulated during the cell cycle in *Crithidia fasciculata* [16] and *T. brucei* [17], however its transcript levels peak some time after those of the *DHFR* co-regulated group. Whether *LIGKA* is unique or a member of a broader group of similarly regulated transcripts is unknown, as is the nature of the RNA elements responsible for its regulation in *cis*. Differential expression may also occur via mechanisms operating at the levels of protein translation, localization and/or stability, since the kDNA ligase proteins are unstable and differentially localized during the cell cycle [16]. Indeed, regulation at both the mRNA and protein levels could synergize to ensure that certain key players in DNA replication, organelle replication, and cell division are tightly regulated. This would especially apply to proteins whose ectopic expression at other points in the cell cycle could short-circuit the program of organellar and cellular duplication.

Since kinetoplastid protozoa rely on RNA-binding proteins, rather than transcription factors, to regulate gene expression, their genomes might be expected to contain a disproportionately large number of genes coding for proteins with RNA-binding domains. This is certainly true for proteins possessing a Puf (Pumilio/Fem-3) RNA-binding domain, of which we found at least 10 encoded in the *T. brucei* genome [18]. The structure of the Puf domain consists of multiple copies of a tri-helical Puf repeat. Each tri-helical repeat binds one nucleotide via three key amino acid residues that cooperatively determine the base preference for that repeat [19],[20]. The various Puf proteins found in yeast bind a significant proportion of all mRNAs and those mRNAs bound by the same Puf protein tend to encode proteins that function in similar locations and processes [21]. Thus, Puf proteins have found functions regulating several large post-transcriptional regulons in a single-celled eukaryote.

Here we describe a Puf protein of *T. brucei*, PUF9, which is conserved within kinetoplastids and possesses 6 copies of the Puf tri-helical repeat. Our results indicate that in mid-to-late S-phase, PUF9 neutralizes a specific destabilizing sequence motif present on its target mRNAs, thus stabilizing them. Consistent with their temporal expression profiles, some of the proteins encoded by PUF9 target mRNAs appear to play roles in maturation and segregation of the daughter kinetoplasts after division, a role supported by the protein localization and over-expression phenotype of an uncharacterized PUF9 target, *PNT1*, as well as the previously reported characteristics of another target, *LIGKA*. A third PUF9 target transcript, *PNT2*, encodes a nuclear protein that relocates to the mitotic spindle midzone during nuclear division. Hence, PUF9 could function in the temporal coordination of nuclear and kinetoplast replication.

## Materials and Methods

### Plasmid constructs and trypanosome culture

All *T. brucei* cells used were derived from the Lister 427 line. To obtain stably transformed clonal lines, 1-2×10^7^ cells were transfected by electroporation with ∼10 µg linearized DNA at 1.5 kV followed by cloning by limiting dilution in medium containing the appropriate selective drug. For tet-inducible expression constructs, expression was induced by including 100 ng/ml tetracycline in the culture medium. Plasmids created for transformation of *T. brucei* cells are summarized in Table 1. Primers used to generate PUF9 fragments by PCR for cloning were as described [18]. The *PNT1* ORF was amplified using the following primers:

GATAAGCTTATGTTGTCCCGAGCCCCA / GATGGATCCGCCGTTCTCACTGCTCACG.

The *PNT2* ORF was amplified using:

GATAAGCTTATGCAGTGGAAGAAAGATGACT / GATGGATCCGAAATGCAGAGGTAAACTTTCG.

The *PNT1* 3′ intergenic region was amplified using:

GATCGGATCCGCATAGATGGAGAGAGTTATACG / GATCACTAGTCTCCACCTTTGTCACTATCCTG.

**Table 1:** Summary of DNA constructs generated.

Construct	Used for	Promoter	Integration target	Selection	Parent construct	Insert
pHD1411	*PUF9* over-expression	*PARP* Tet Op	rDNA intergenic	Hyg	pHD615	*PUF9* ORF
pHD1456	TAP tagged PUF9	*PARP* Tet Op	rDNA intergenic	Hyg	pHD918	*PUF9* ORF
pHD1876 –1880	CAT reporters	rRNA	rDNA intergenic	Puro	pHD1034	*PNT1* 3′ intergenic region
pHD1888 pHD1966	CAT reporters	–	*tubulin* intergenic	Puro	pHD1887	*PNT1* 3′ intergenic region
pHD1860	*PUF9* hairpin (RNAi)	*PARP* Tet Op	rDNA intergenic	Hyg	pHD1146	*PUF9* ORF fragment
pHD1489	*PUF9* dsRNA (RNAi)	2x T7 Tet Op	177 bp repeat	Hyg	p2T7-177 [46]	*PUF9* ORF fragment
pHD1985	Myc-tagged PNT1	*PARP* Tet Op	rDNA intergenic	Hyg	pHD1700	*PNT1* ORF
pHD2005	Myc-tagged PNT2	*PARP* Tet Op	rDNA intergenic	Hyg	pHD1700	*PNT2* ORF

Plasmids generated here to express transgenes in *T. brucei*. Indicated is the promoter upstream of the transgene cassette (tetracycline-inducible promoters are indicated with Tet Op); the integration target (the genomic sequence into which the construct should be targeted by homologous recombination); the resistance marker genes used to select for transformants (conferring either hygromycin or puromycin resistance); the parent construct and the inserted sequence. Further details of the construction and sequences of these plasmids are available upon request.

### Site-directed mutagenesis

Point mutations were introduced into the *PNT1* 3′ UTR sequence in CAT-reporter plasmid pHD1876 by site-directed ligase-independent mutagenesis (SLIM) [22] in a multiplex PCR reaction using a plasmid containing the wild-type UTR as template and the forward primers:

GTAATGTAACATTATACCATTTGTGTTGTTGTTTAG and ACCATTTGTGTTGTTGTTTAG

and reverse primers:

T AATGTTACATTACAACACCCGCTGCAGAATTTTTGTG and ACCCGCTGCAGAATTTTTGTG

(mutated residues underlined). The products of this reaction were heat-denatured, re-annealed and transformed into *E. coli*. Plasmids from the resulting transformants were isolated and sequenced to check for side-mutations. Due to a strain-specific G→T SNP in the Lister427 gDNA initially used as template, the G residue 9 nt downstream from the point mutation is actually a T in the wild-type *PNT1* 3′ UTR but was mutated back to a G in this point-mutant construct, because the SLIM primers were designed from the published genomic sequence from the TREU 927 strain.

### TAP immunoprecipitation

The TAP (Tandem Affinity Purification) tag used here possesses Protein A and Calmodulin Binding Protein domains. The *PUF9* ORF was cloned into plasmid pHD918, generating a construct encoding PUF9 linked to the TAP tag at the C-terminus via a peptide linker that contains a TEV protease cleave site. This was expressed from the *PARP* promoter, under the control of the Tet-repressor, in bloodstream form (BS) cells by induction with 100 ng/ml tetracycline for 24 hr. RNA co-purification was performed as described [23]. Approximately 3×10^9^ cells were induced to express the fusion protein by the addition of tetracycline for 12–24 hr prior to harvesting. Cells were washed in cold PBS, crosslinked on ice by UV irradiation at 400 mJ/cm^2^ in a Stratalinker, then snap frozen. Cell pellets were broken in 6 ml breakage buffer (10 mM Tris-HCl pH 7.8, 10 mM NaCl, 0.1% IGEPAL CA 630 (Sigma; identical to the previously used detergent Nonidet P-40), 4 mM Vanadyl Ribonucleoside complexes (VRCs, Sigma), 4 U/ml RNAseIn (Promega), 1× Complete Inhibitor without EDTA (Roche)) by passing through a 21-gauge syringe 15 times at 4°C. Insoluble material was removed by ultracentrifugation (100,000 ×g, 45 min at 4°C) and the salt concentration of the supernatant was adjusted to 150 mM. 200 µl of IgG sepharose bead suspension (Fastflow – GE Healthcare) was washed in IPP150 and rotated with the lysate for 2 hours at 4°C. IPP150 contained 10 mM Tris-HCl, pH 7.8, 150 mM NaCl, 0.1% IGEPAL CA 630. The flow-though was collected, and beads washed three times in 10 ml of IPP150 and once in 10 ml of TEV cleavage buffer (IPP150 with 0.5 mM EDTA, 1 mM DTT, 2 mM VRCs, 4 U/ml RNAseIn (Promega)). The TAP tag was then cleaved by adding 1 ml of TEV cleavage buffer and 100 units of TEV protease (Invitrogen), and rotating the beads for two hours at 16°C followed by collecting the eluate. RNA was isolated from the eluate using the QIAgen RNAeasy kit or Trizol LS according to the manufacture's instructions. The entire procedure was scaled down if less RNA was required, *e.g.* for RT-PCR. Aliquots, equivalent to 4×10^6^ cells, were taken at various points in the procedure for analysis by western blot. Calmodulin selection was not used for RNA isolation due to the requirement to add calcium to the buffer during binding.

### Microarray analysis

Genomic *T. brucei* microarrays were generated containing 24,567 random shotgun clones from *T. brucei brucei* strain TREU927/4 genomic DNA [9]. Test and control samples of RNA were reverse-transcribed using SuperscriptII (Invitrogen) according to the manufacturer's instructions in the presence of either Cy5-dCTP or Cy3-dCTP and cDNA purified using the QIAquick PCR purification kit (QIAGEN), ethanol-precipitated and resuspended in 5 µl TE. Cy3- and Cy5- labelled cDNAs were mixed, denatured at 95°C for 5 min and snap-chilled, then added to 60 µl of hybridization buffer (50% formamide, 3× SSC, 1% SDS, 5× Denhardt's reagent and 5% dextran sulphate). This was added to the slide, a coverslip affixed and incubated at 62°C overnight in a humidified chamber. Slides were washed at RT for 10 min in 2× SSC, 0.2% SDS, 10 min in 2× SSC, and 10 min in 0.2× SSC, dipped in isopropanol and dried. Microarrays were scanned with ScanArray 5000 (Packard BioScience, Dreieich, Germany) and analyses of resulting images were performed using GenePix software (Axon Instruments, Union City, USA). The software package MCHIPS [24] was used for data quality assessment and normalization. Clones corresponding to positive hits were sequenced from one end and mapped onto the published *T. brucei* genome.

### RNA blotting

For RNA detection by Northern blot, RNA was size-separated by overnight agarose-gel electrophoresis on a 3.5% formaldehyde gel, transferred onto a nylon membrane by capillary transfer and fixed by UV irradiation as described [25]. The membrane was prehybridized in a hybridization bottle in 5× SSC, 0.5% SDS with salmon sperm DNA (200 µg/ml) and 1× Denhardt's solution for 2 hours at 65°C. Probe was generated by PCR in the presence of [32P]-labelled dCTP followed by purification using the QIAGEN nucleotide removal kit according to the manufacturer's instructions. Probe was added to the prehybridization solution and the bottle rotated at 67°C overnight. After rinsing the membrane in 1× SSC/0.5% SDS, probe was washed out with two 20 minute washes in 0.2× SSC/0.5% SDS at 67°C and the membrane exposed on a phosphorimaging screen for 2–48 hours. The screen was read on a recently calibrated Fugifilm FLA-3000 reader. Signal density from bands were quantified in Image Quant v3.45 and background signal density from a nearby region within the same lane of the gel was quantified and subtracted from the value for the band. Blot picture intensities were adjusted such that the darkest pixel was set to zero intensity, and 5% of the lightest pixels were clipped to 100% intensity.

### Semi-quantitative RT-PCR

TAP-copurified RNA, or RNA from the flow-through (derived from the equivalent of ∼2×10^8^ or 4×10^7^ cells respectively) was reverse-transcribed using a cocktail of gene-specific primers and Superscript III reverse transcriptase (Invitrogen) in a 20 µl reaction. This was used as template in a semi-quantitative PCR reaction to detect control and test genes (1 µl cDNA per 50 µl reaction). Samples (7 µl) were removed after 28, 32 and 36 cycles and analysed on an agarose gel. All primer pairs were designed using Primer3 [26] and had similar melting temperatures (∼60°C) and product lengths (250–400 nt).

### Synchronization of procyclic cells

We adapted the cell-starvation protocol previously described [27], which produces semi-synchronous cultures arrested predominantly at the G1 phase by starvation. Cells were seeded at 1×10^6^/ml, and two days later the starved culture (∼2–4×10^7^ cells/ml) was diluted in auto-conditioned MEM-pros medium [28] to induce resumption of the cell cycle. Aliquots of ∼10^6^ cells were taken at regular intervals over the next 9–17 hours for flow cytometry. Cells were collected from these samples by centrifugation, resuspended in 100 µl of PBS, fixed by dropwise addition of 1 ml of 70% ethanol, 30% PBS while gently vortexing, and stored at 4°C. Cells were collected by centrifugation (2000 ×g, 10 min), resuspended in 500 µl of PBS with 10 µg/ml RNase A and 30 µg/ml propidium iodide, incubated at 37°C for 30 minutes and analysed by FACSSCAN. The proportion of cells in each phase of the cell cycle was estimated using the Watson algorithm [29] as implemented in the Flowjo software package.

### Immunofluorescence staining

Approximately 10^6^ cells were collected by centrifugation and resuspended in 50 µl of PBS. Cells were fixed in 4% paraformaldehyde (or 4% formaldehyde/5% acetic acid if staining for mitotic spindles) in PBS for 20 min, washed twice in PBS and allowed to settle onto polylysine-coated slides. Cells were permeabilized in 0.1% Triton-X in PBS, washed twice and blocked in 1% BSA or gelatine for 1 hour. Primary antibody was added at the recommended dilution, incubated for 1 hour and the slides washed 3 times in PBS before addition of fluor-conjugated secondary antibody in the dark for 1 hour. Slides were washed, stained with DAPI for 10 minutes, and washed twice more before drying the slide. A drop of Vectorshield was added, the coverslip was affixed, and cells were viewed on a Leica DM fluorescence microscope. Where Mitotracker staining was used, Mitotracker CMXros (Invitrogen) was added to the cell culture medium at 250 nM for 30 min, after which the cells were pelleted and resuspended in fresh media. After incubating for 15 minutes, cells were fixed and stained using the protocol described above.

### Bioinformatics analysis

The 3′-UTR sequences of *T. brucei* genes were taken from the published genomic sequence of the TREU 927 strain, using start and end positions predicted previously [30] or, where the prediction was absent, taking the entire intergenic region to a maximum of 5 kb downstream of the stop codon. The sequences of interest were compared to 1000 randomly chosen 3′ UTRs as a background sample using Trawler [31]. Homologous genes in *Trypanosoma congolense* were also identified by TBLASTN searches and the 3′-UTR sequences predicted from the downstream intergenic regions by similar means.

### RNA half-life assay

Synthesis and maturation of mRNA were simultaneously inhibited by addition to the growth medium of 10 µg/mL actinomycin D and 2.5 µg/ml sinefungin. Sinefungin was added 5 minutes prior to actinomycin D [32]. Cells were collected at the indicated time points and RNA isolated by Trizol extraction. RNA levels were estimated by Northern blotting using [32P]-labelled probes, and quantitated by phosphorimaging. The stable *SRP* RNA was used as a loading control.

## Results

### *PUF9* RNAi causes an accumulation of G2-phase cells

**Figure 1:**
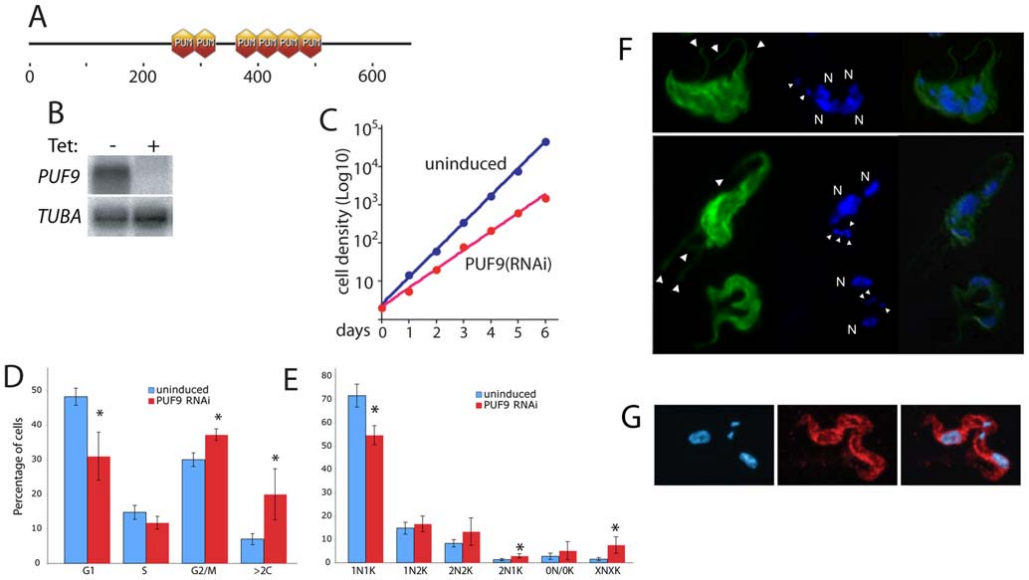
PUF9 structure and RNAi phenotype. A: Predicted domain structure of PUF9. Output from ExPASY showing six predicted Puf domains in the central region of the protein. B: Northern blot of RNA from BS cells, showing tet-inducible RNAi against the native ∼6 kb *PUF9* transcript, normalized using the *TUBA* transcript. C: Cell densities of BS cultures were monitored with or without *PUF9* RNAi. Cells were counted and diluted to 5×10^4^/ml every 24 hours. A cumulative curve is shown. Calculated generation times are indicated. D, E: Cells were scored for DNA content using flow cytometry (D), and for nuclear/kinetoplast division status by DAPI-staining and fluorescence microscopy (E) in *PUF9* RNAi BS cells. RNAi was induced with tetracycline for 24 hours. At least 200 cells from each of four independent experiments were counted (microscopy). Cells lacking either the kinetoplast or nucleus were scored as 0N/0K while cells with >2 kinetoplasts or nuclei were scored as XNXK. The 95% confidence intervals are shown. (*) indicates significant difference from untreated controls by Student's T-test (*p*<0.05). F: Immunofluorescence microscopy on *PUF9* RNAi BS cells. Cells were fixed and stained for β-tubulin with the KMX-1 antibody (a gift from Keith Gull) and for DNA with DAPI. Cells at top and middle are large (magnification for all panels is the same) and possess multiple flagella (large arrowheads), kinetoplasts (small arrowheads) and nuclei (N). At the bottom are two normal cells. G: C-terminally TAP-tagged PUF9 protein was localized to the cytoplasm by immunofluorescence using antibody against protein A (red). Nuclei and kinetoplasts were stained using DAPI (blue).

The Puf domain protein PUF9 (Tb927.1.2600) is conserved among *Trypanosoma* and *Leishmania* species. It contains 6 Puf repeats, as well as extended N- and C- terminal domains lacking any homology to characterized proteins (Figure 1A). We decided to investigate the biological function of PUF9 through RNAi and by co-purification of target RNAs.

The phenotype of bloodstream-form (BS) cells in which PUF9 was depleted by tet-inducible RNAi, or over-expressed using a tet-inducible VSG promoter, was examined. A Northern blot confirmed *PUF9* mRNA knockdown after 24 hours of induction (Figure 1B). Although over-expression of *PUF9* did not cause any noticeable phenotype, *PUF9* RNAi reduced overall cell growth over the six days of RNAi induction (Figure 1C). We used freshly thawed clonal cell lines, which may account for the growth phenotype not seen previously [18]. Flow cytometry revealed an accumulation of cells with 2C DNA content (G2/M cells) in *PUF9* RNAi cells relative to uninduced cells (Figure 1D), and there were also more polyploid (>2C) cells. Examination of trypanosomes by fluorescence microscopy after staining for DNA can also be used to score them for cell cycle stage: cells with a single nucleus and kinetoplast (1N1K) are in G1 or S phase, cells with two kinetoplasts and one nucleus (1N2K) are in G2, and cells with two kinetoplasts and two nuclei (2N2K) are mitotic or post-mitotic. The proportion of 1N1K cells was lower in *PUF9* RNAi cells (Figure 1D). In addition, the *PUF9* RNAi cells often possessed more than two flagellae, nuclei, or kinetoplasts (Figure 1E). Extra nuclei or kinetoplasts were seen after 24 hours of *PUF9* RNAi (Figure 1D), suggesting a possible defect in control of organelle copy number. However, there was no obvious difference in the occurrence of annucleate “zoid” cells. Similar experiments in insect-form procyclic (PC) cells yielded no obvious phenotype (not shown).

### Four mRNAs co-enrich with TAP-tagged PUF9

In order to find out which mRNAs were targeted by PUF9, we expressed the protein with a C-terminal TAP tag in BS cells to allow affinity purification. Western blotting indicated that the tagged protein was stable in BS cell lysate and it was found in the cytoplasm of BS cells by immunofluorescence staining (Figure 1F). Protein-RNA complexes from BS lysates were selected on an IgG column, and eluted by cleaving the tag with TEV protease. Cells expressing the TAP tag alone served as a control. RNA that co-precipitated with PUF9::TAP or the TAP-tag alone was reverse transcribed with fluorescently labelled nucleotides and the cDNA hybridized to a microarray of shotgun genomic clones. Spots showing more than 2-fold higher intensity for the PUF9 channel were flagged and the corresponding genomic clones end-sequenced. Results from two biological replicates are summarized in Table 2. Several genomic loci were identified from multiple overlapping DNA clones, indicating that a gene of interest was present in the overlapping regions. Genes were considered as candidates for interactions with PUF9 if more than one spot was flagged that contained sequences overlapping that gene, or the same spot containing the gene was flagged from both biological replicates. rRNA and *PUF9* itself were also identified in one replicate. While intriguing, it is possible that these hits may represent artefacts caused by the over-expression of the tagged PUF9, which is integrated into an *RRNA* locus, leading to higher background levels of these two RNAs. The number of transcripts that were identified was surprisingly small in comparison to similar experiments in yeast where hundreds of transcripts were coprecipitated with PUF proteins [21]; nonetheless, the fact that sequencing of several different flagged spots resulted in them repeatedly being mapped back to the same few transcripts indicates that significant coverage of high-affinity PUF9 targets was attained.

**Table 2:** Identification of PUF9-copurifying RNAs by microarray.

Spot probe	Replicate 1	Replicate 2	Gene(s) present on spot probe	Locus group
05P19	X	X	***Tb11.02.4400 (PNT1)***	1
32E16	X	X	***Tb11.02.4400 (PNT1)***	1
35F01	X	X	***Tb11.02.4400 (PNT1)***	1
22P10	–	X	***Tb11.02.4400 (PNT1)*** * Tb11.02.4390*	1
22D07	X	X	*Tb11.01.6460 Tb11.01.6450*	2
39O15	–	X	***Tb11.01.6470 (PNT2)*** * Tb11.01.6480*	2
55O18	–	X	***Tb11.01.6470 (PNT2)*** * Tb11.01.6460*	2
16L12	–	X	*Tb927.7.600 (LIGKB )*	3
32F10	X	X	***Tb927.7.610 (LIGKA)***	3
58C08	X	X	***Tb927.2.2670 (H4V)***	–
40D05	X	X	*rRNA*	–
48G14	–	X	*Tb10.70.0220*	–
21N07	–	X	*Tb927.1.2600* (*PUF9*)	–
31G22	–	X	*13J3.10 (ESAG6) 13J3.11 (hp)*	–
52L23	–	X	*Tb927.5.2560*	–

RNA co-precipitating with the PUF9::TAP fusion (specifically co-purifying) and the TAP tag-only control (non-specifically co-purifying) was isolated and cDNA was generated, incorporating either Cy3 or Cy5 dyes. The two samples were compared by hybridization with a microarray of shotgun genomic clones. Two biological replicates were performed, and probes showing greater intensity for the PUF9 channel are marked “X”. Probes of interest were end-sequenced and their internal sequences estimated by comparison with genomic sequence data. Probes were grouped by locus (right column) in cases were multiple positive probes were mapped to overlapping or adjacent genomic regions. Genes that were further tested as candidate PUF9 targets are shown in bold.

**Figure 2:**
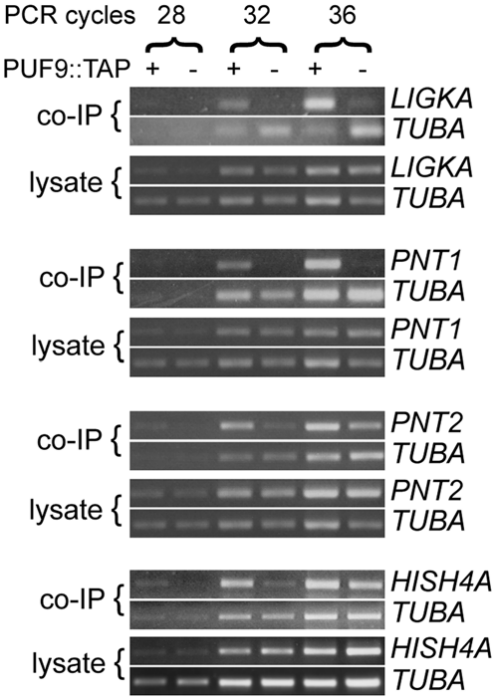
Verification of candidate PUF9 target transcripts. RNPs were isolated by TAP purification from lysates of BS cells expressing the PUF9::TAP fusion protein (+) or the TAP tag alone (−). RNA was purified from the affinity-purified product (co-IP) and the flow-through fractions (FT) and used as templates for reverse transcription with a cocktail of gene-specific primers. The cDNA was used as template in PCR reactions to detect the indicated genes, and *TUBA* was used as an internal control for template concentration. Samples were collected at 28, 32 and 36 cycles, and analysed by agarose gel electrophoresis with ethidium bromide staining. No bands were detected when reverse transcriptase was omitted (not shown).

Since some microarray hits spanned several adjacent genes, another independent TAP-purification was performed and the co-purifying RNA was analysed for enrichment of mRNAs by semi-quantitative RT-PCR using primers specific for individual gene ORFs. Four candidate sequences were amplified more strongly from the PUF9-copurified RNA than the TAP-only copurified RNA, while amplification of the abundant *TUBA* transcript was approximately equal between the samples (Figure 2). This was not due to differences in transcript abundance in the lysates since RT-PCR on RNA isolated from flow-through fractions showed no detectable enrichment. The confirmed PUF9-associated transcripts are *LIGKA* (kDNA ligase α/*Tb927.7.610*), a histone H4 variant (*H4V*/*Tb927.2.2670*, possibly involved in transcription termination [33]), and two uncharacterized kinetoplastid-specific genes that we have named Puf Nine Target 1 (*PNT1*/*Tb11.02.4400*), and Puf Nine Target 2 (*PNT2*/*Tb11.01.6470*). *Tb11.02.6460*, an adjacent gene to *PNT2*, was also tested (not shown) because we could not delineate which of the two transcripts was responsible for the hits around this genomic locus using the microarray data alone (Table 2). However, only *PNT2* mRNA was found to be a genuine target of PUF9 after analysis by RT-PCR. The *PUF9* transcript itself could not be amplified from cDNA from the PUF9-copurified RNA. However, this transcript has an exceptionally long 3′ UTR that might be vulnerable to degradation during the procedure.

### PUF9 stabilizes target transcripts

**Figure 3:**
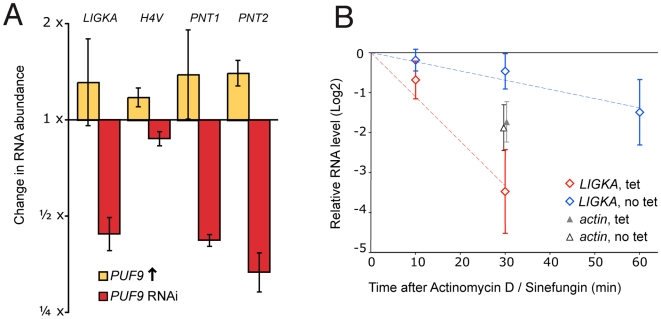
Effects of PUF9 on abundance and stability of target mRNAs. A: Relative expression of PUF9 target mRNAs with alterations in *PUF9* expression. *PUF9* over-expressing or *PUF9* RNAi BS cells were uninduced or induced with tet for 24 hours prior to RNA isolation and Northern blotting. The blot was probed for *PUF9* and PUF9 target genes, and transcript abundance was determined by densiometric quantification. All bands were normalized against *TUBA* and then normalized against their abundance in the uninduced controls. Mean and standard error (*p* = 0.05) from three biological replicates are shown. B: Quantification of *LIGKA* mRNA after inhibition of RNA synthesis and processing. *PUF9* RNAi or uninduced BS cells were treated at time t = −5 min with sinefungin and at time t = 0 with actinomycin D to inhibit mRNA processing and transcription, respectively. RNA was isolated at the indicated times and the *LIGKA* transcript was quantified by Northern blot and densiometric scanning on a phosphorimager. Band intensity was normalized against background and the stable *SRP* RNA was used as a loading control. Values were expressed relative to the intensity at t = 0 which was fixed at 1. Five biological replicates per condition were performed and used to generate a line of best fit on the log-transformed values. Three of these were also probed for the actin transcript as a control (normalized values at t = 30 shown as triangles). Error bars represent standard error of the mean (*p* = 0.05). Bands from the t = 60 time point were excluded as they were too faint to accurately quantify, except for the *LIGKA* transcript in the uninduced cells.

The effect of PUF9 on its target transcripts was examined by Northern blotting of RNA from the *PUF9* over-expressing and *PUF9* RNAi BS cells. Probing the Northern blots for the four target genes showed that RNAs from three of them were clearly more abundant when PUF9 was over-expressed and less abundant when PUF9 was depleted (Figure 3A and Figure S1), while the remaining target, *H4V*, was only slightly affected. Because *H4V* transcript is only weakly regulated by PUF9, it was excluded from further analysis here. However, its association with PUF9 could still indicate regulation at a different level, *e.g.* translation, that was not tested in this work. *Tb11.01.6460*, which is adjacent to *PNT2*, showed no dependence on PUF9 (not shown), consistent with the PUF9-mediated upregulation operating on individual mature transcripts rather than genomic loci or polycistronic precursor RNA.

To find out whether mRNA half-lives were influenced by PUF9, we inhibited mRNA synthesis using actinomycin D and sinefungin [32], and followed the abundance of the PUF9 target mRNA *LIGKA*. Actinomycin D binds to DNA and inhibits RNA transcription elongation, while sinefungin inhibits methylation of the spliced leader RNA leading to a blockage in mRNA maturation [34]. *LIGKA* mRNA half-life was approximately 30 minutes in normal cells, but was reduced four-fold when PUF9 was depleted by RNAi, while the half-life of the actin control transcript remained unchanged (Figure 3B). These data support a role for PUF9 in stabilizing its target mRNAs. The effects on target transcript abundance and half-life were not due to slower growth or the previously observed increase in the proportion of cells in G2 phase, because they also occurred when *PUF9* was targeted by RNAi in PC cells, which exhibited wild-type growth and flow cytometry profiles (see below).

### Abundance of PUF9 target transcripts varies during the cell cycle

The PUF9 target gene *LIGKA* has a homologue in *C. fasciculata* (kinetoplast DNA ligase α), for which the mRNA was previously shown to vary in abundance with the cell cycle [16]. This, together with the *PUF9* RNAi phenotype that hinted at a defect in cell cycle progression, led us to investigate whether PUF9 plays a role in cell-cycle-coupled differential expression of genes. PC cells are amenable to synchronization by starvation [27] and by hydroxyurea treatment [35] while BS cells have also recently been synchronized by hydroxyurea [36]. Although no hydroxyurea-generated artifacts have been observed during synchronization of *T. brucei*, drug-mediated synchronization has been observed to cause uncoupling of DNA replication status from cyclin levels in other systems [37], therefore we opted to follow the *T. brucei* starvation-induced synchronization protocol [27].

**Figure 4:**
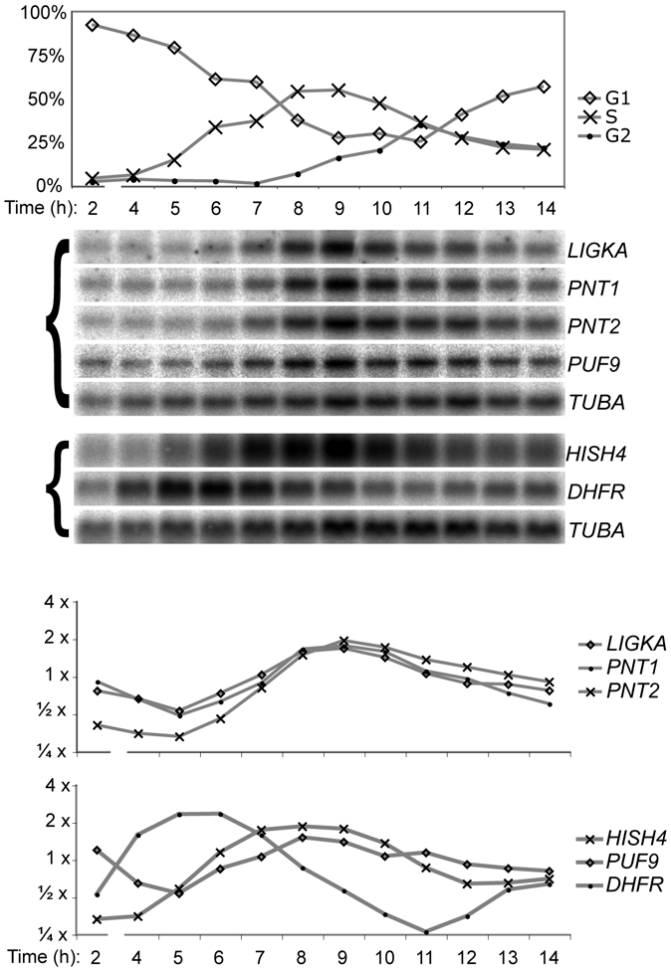
Cell-cycle-coupled regulation of PUF9 target transcripts and the *PUF9* transcript itself. Cells were synchronized in G1 phase by starvation for 2 days and released by dilution in fresh media. Samples were taken every hour for flow cytometric analysis of cell cycle phase (top) and also for RNA isolation and Northern blotting. All data are from a single representative experiment. Top: The percentage of cells in each phase of the cell cycle, calculated from flow cytometry histograms using the Watson algorithm. Center: Duplicate Northern blots (indicated with a left bracket) from the synchronized cells were probed for the three PUF9 targets and *PUF9*, as well as *DHFR* and the histone *HISH4* as controls for synchronization and *TUBA* was used as a loading control. Bottom: Quantification of transcript abundance by densiometric scanning of the Northern blot, normalizing with respect to *TUBA* mRNA.

Starved PC trypanosomes accumulate in the G1 phase of the cell cycle (Figure 4). Upon release from starvation, about 70% of cells begin to progress through the cell cycle after a ∼4 hour lag (Figure 4). Analysis by Northern blot showed that transcript levels of housekeeping genes such as *TUBA* increased rapidly for the first hour, then increased at a steady rate throughout the assay, relative to structural RNAs such as *SRP* (data not shown), therefore *TUBA* was used as a “baseline” mRNA transcript to normalize for loading and the global effects of starvation upon mRNA levels. *DHFR* and *HISH4* transcripts, which are known to be regulated during the eukaryotic cell cycle, peaked in early- and mid- S-phase respectively, consistent with good synchronization of the recovering cell culture (Figure 4). The timing of their transcript maxima fits with the fact that DHFR protein is needed prior to DNA synthesis while HISH4 is required during or immediately after synthesis. The three PUF9 target genes *LIGKA*, *PNT1* and *PNT2* were also regulated during the cell cycle, peaking in mid- to late- S-phase. The *C. fasciculata* homologue of *LIGKA* also cycles out-of-phase with the *DHFR* transcript in that organism [16].

### PUF9 mediates upregulation of target transcripts in S-phase

**Figure 5:**
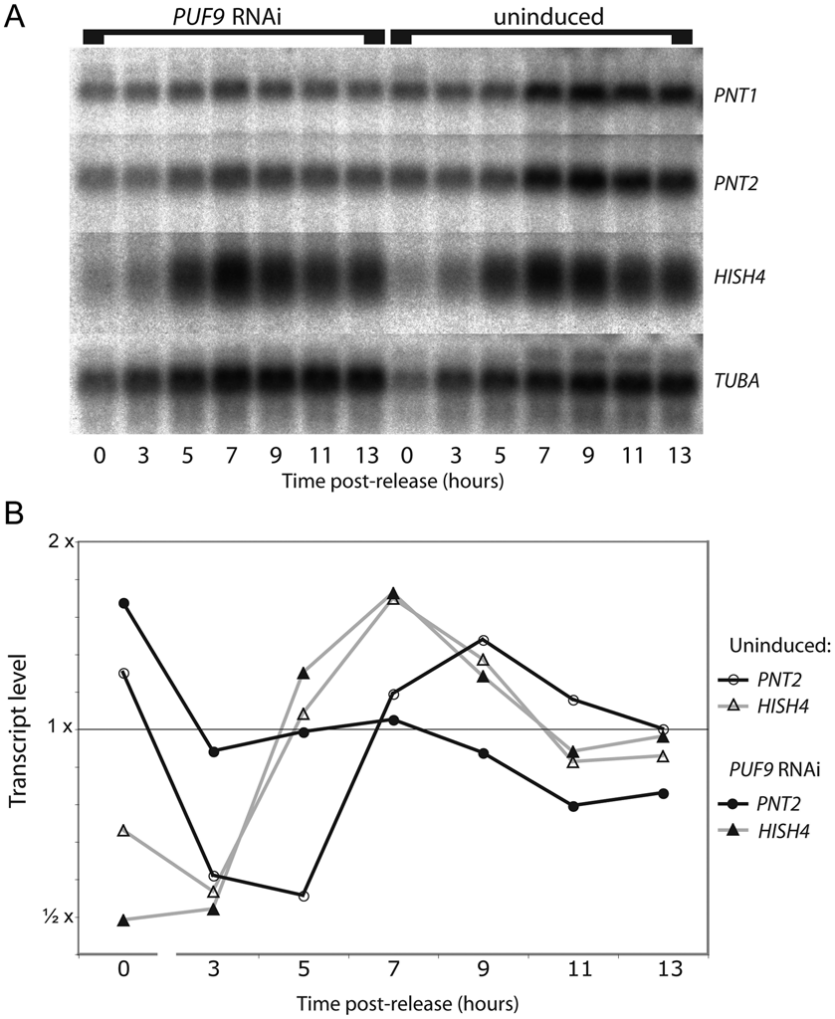
Effect of *PUF9* RNAi on oscillation of its target transcripts in the cell cycle. A: PC cells were induced to express a hairpin transcript to target *PUF9* for RNAi during starvation-mediated G1 synchronization. After release by dilution in fresh media, samples were taken every two hours for flow cytometry to check for synchronization (not shown) and for isolation of RNA for Northern blotting. The Northern blot was probed for the PUF9 target *PNT2*, as well as *TUBA* to normalize for loading and *HISH4* as a control for synchronization efficiency. B: Normalized quantification of the data shown in A. For all transcripts, the average intensity throughout the assay for that condition was set to 1.

To determine whether PUF9 plays a role in the S-phase-specific upregulation of its target genes, *PUF9* was targeted by RNAi in procyclics during synchronization. Northern blotting showed that RNAi against *PUF9* effectively repressed the *PUF9* transcript and also lowered levels of PUF9 target transcripts as with BS cells (Figure S2); although as previously noted, *PUF9* RNAi had no effect on PC growth, perhaps due to residual expression that is sufficient for function in PCs, or stage-specific differences between the cell-cycle checkpoint mechanisms. Flow cytometry also showed that there was no significant difference between synchronization efficiencies of induced and uninduced *PUF9* RNAi PC cells, and this is supported by the nearly identical cyclical expression of the cell-cycle regulated *HISH4* transcript between induced and uninduced cells (Figure 5A, triangles in 5B). However, probing for the *PNT1* and *PNT2* transcripts revealed that they no longer oscillated over the cell cycle when RNAi against *PUF9* was induced (Figure 5A, circles in 5B), indicating that PUF9 is required for the peak in their transcript levels that occurs in S-phase in the control cells.

Interestingly, the *PUF9* transcript itself also showed moderate cell cycle-coupled regulation, peaking at a similar time to its target mRNAs (Figure 4 bottom panel). However, it seems doubtful that this relatively moderate regulation at the mRNA level could fully account for the larger changes in expression of the target mRNAs, so it is likely that other regulatory mechanisms exist. Attempts to generate PUF9 antisera failed; therefore we tagged one *PUF9* allele *in situ* with an N-terminal V5 epitope. However, the protein showed approximately the same degree of variation through the cell cycle as was previously observed for the mRNA levels, and no difference was seen in the electrophoretic mobility by western blot (not shown). It should be noted that N-terminal *in situ* tagging replaces the 5′ UTR, so 5′ UTR-dependent translational regulation would not be detected. We also attempted to co-purify interacting protein partners of PUF9 by tandem affinity purification of the TAP-tagged PUF9 protein, but mass-spectrometry of affinity-purified protein bands only revealed degradation products of PUF9 itself. Thus, the mechanism whereby PUF9 specifically stabilizes its target transcripts in late S-phase remains to be elucidated.

### Identification of a cell-cycle response motif in target transcripts

**Figure 6:**
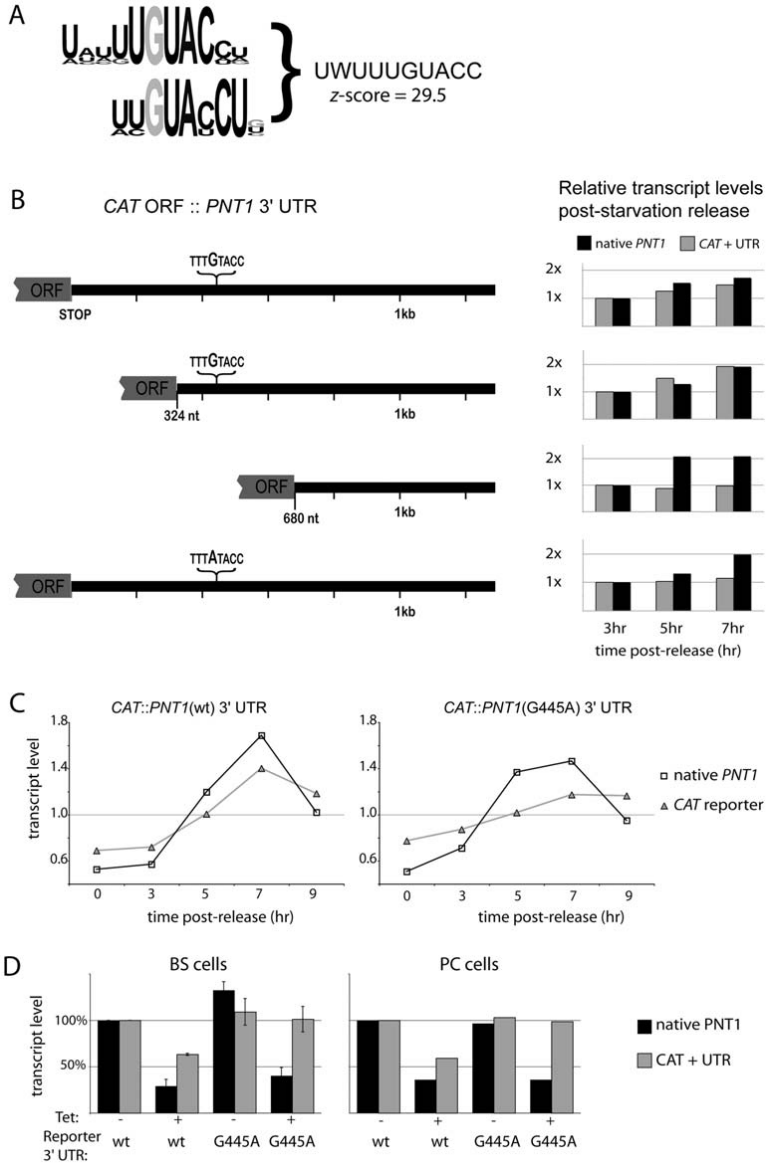
Identification of regulatory elements in PUF9 target mRNAs. A: The over-represented motif identified by Trawler. B: Derivatives of the *PNT1* 3′ UTR were fused to the *CAT* reporter ORF and tested for cell cycle-coupled transcript regulation. Quantification of Northern blots of synchronized cell cultures are shown. At least one replicate experiment was performed for each reporter shown, yielding similar results. Transcripts were normalized against *TUBA* and then against their own values at 3 hours post-release. C: Quantification of *CAT* reporter transcript abundance, fused to the *PNT1* 3′ UTR or the derived G445A point mutation, during progression through one cell cycle. Transcripts were normalized against *TUBA* and then against their own values at 3 hours post-release. D: Dependence of reporter transcript stability on PUF9 expression. The reporters described above (wt or G445A) were transformed into PC cells containing the *PUF9* hairpin construct and RNAi against *PUF9* was induced for 16 hours. RNAi was confirmed by Northern blot and probing for the native *PNT1* transcript, which is dependent on PUF9 expression. Reporter transcript abundance was revealed by probing for the *CAT* ORF and normalizing against *SRP*. All transcripts were then normalized against their own abundance in the wt UTR/uninduced condition. Error bars represent 95% confidence intervals.

PUF9 targets appear to be regulated independently to most previously characterized cell-cycle-regulated transcripts (*e.g. DHFR*, *TOP2*, *RPA1*), which peak at a different time and possess known sequence motifs that act as cell-cycle regulatory elements (CCREs) [10],[11],[12],[13],[16]. To identify the RNA determinants responsible for PUF9-mediated cell-cycle-coupled transcript regulation, a combination of bioinformatics and experimental approaches was used. The 3′ UTRs of the three known mRNAs regulated by PUF9 were analyzed using Trawler, a program that identifies over-represented motifs in sets of sequences relative to a background set of genes [31]. This strategy is useful for identifying putative recognition sequences for Puf proteins because they tend to recognize primary RNA sequences rather than secondary RNA structures [38]. The most over-represented motif instance identified by Trawler was “UUGUACCW”, found 7 times in the 3 sequences. The best cluster within this family is summarized in a position weight matrix (Figure 6A). This motif is a promising candidate PUF9-recognition motif as it contains the Puf protein consensus core binding sequence “UGUA” [39]. In addition, the predicted key nucleotide-binding residues of the 4^th^, 5^th^ and 6^th^ Puf repeats in the *T. brucei* PUF9 protein are homologous, respectively, to “U”, “G” and “U”-binding repeats of characterized Puf proteins [40], indicating that at least the minimal conserved “UGU” trinucleotide forms part of the PUF9 recognition motif. A search of the preliminary *T. congolense* genome (the closest relative of *T. brucei* for which a largely complete genomic sequence is available) shows that it possesses clear homologues to all three *T. brucei* PUF9 targets. Despite being sufficiently distant from *T. brucei* to have no detectable similarity in most of the 3′ UTRs, the three PUF9 target homologues possessed several copies of the candidate motif in their 3′ UTRs.

To test experimentally whether the 3′ UTRs of PUF9 target genes contain CCREs, the 3′ intergenic region of *PNT1* was cloned downstream of a *CAT* reporter gene and the reporter was expressed in procyclic cells. This 3′ UTR was chosen because its three “UGUA” motifs are clustered within a 20 nt region, the last one of which is contained within “UUGUACC” (the motif we identified as being over-represented in PUF9 targets). Cell synchronization experiments showed that the 3′ UTR could indeed confer cell cycle-coupled regulation upon the reporter transcript (Figure 6B, 6C), similar to the behavior of the native *PNT1* transcript, although the magnitude of regulation was somewhat reduced and the peak in transcript level is delayed (see below). This is possibly due to increased stability conferred by the *CAT* ORF or the exogenous 5′ UTR. Alternatively, the 3′ UTR of *PNT1* may contain only one component of a set of dispersed functional elements located over the entire transcript that cooperatively lead to efficient cell cycle-driven regulation. To locate this element, we further examined the *PNT1* 3′ UTR. All the “UGUA” motifs are clustered around ∼425 nt downstream of the stop codon, and initial mapping experiments showed that the region between +324 nt and +680 nt was critical for cycling of the *CAT::PNT1-3′ UTR* reporter transcripts (Figure 6B). We then mutated the central, conserved “G” of the last motif to an “A” and found that this also abolished its ability to confer cell-cycle regulation to the *CAT* reporter transcript (Figure 6B, 6C). Thus, this motif seems to act as a CCRE component. Despite this, preliminary expression-profiling experiments on synchronized cells (data not shown) showed no evidence for cell-cycle regulation in several hundred other transcripts containing at least one copy of this motif in the predicted 3′-UTR region, indicating that, although necessary, it is not sufficient to mediate cell-cycle regulation. Hence, the secondary structural context of the motif or the presence of other cooperating elements may heavily influence its effectiveness.

When the 3′-UTR reporter constructs were transformed into *PUF9* RNAi PC or BS cells, the abundance of the reporter mRNA bearing the wild-type 3′ UTR was dependent upon the expression of PUF9 (Figure 6D). Subsequently, Western blotting indicated that CAT protein levels were also reduced in *PUF9* knockdown cells, but as this seemed to be roughly proportional to the drop in mRNA levels (Figure S3), it is still uncertain whether PUF9 appreciably modulates translation. More elaborate kinetic studies and reporter transcripts also bearing the 5′ UTR of *PNT1* would be required to thoroughly investigate potential translational regulation by PUF9.

Significantly, the same point mutation that abolished cell-cycle response also abolished the dependence of the transcript levels on PUF9 (Figure 6D). This may indicate that the CCRE is potentially a direct binding site for PUF9, and that the point mutation abolishes PUF9 binding. However, if PUF9 were the only player in regulation then the mutant transcript should be constitutively unstable, which is not the case: steady-state levels of the mutant reporter (normalized against the *SRP* RNA) were closer to those of the wild-type in the presence of PUF9.

### Localization and over-expression phenotypes of PUF9 targets

**Figure 7:**
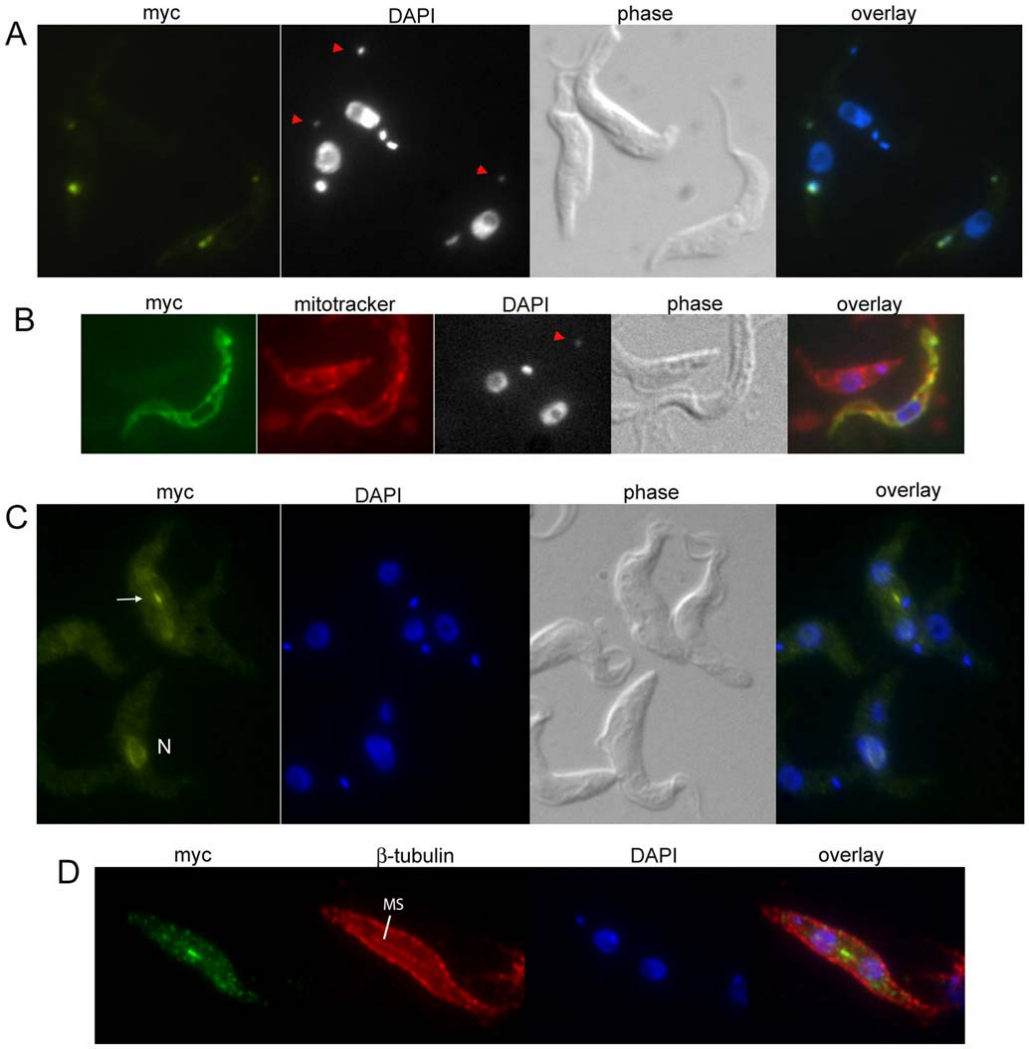
Localization of myc-tagged PNT1 and PNT2 protein. PNT1::myc (A and B) and PNT2::myc (C and D) over-expressing PC cells, induced with tet for 16–24 hours, were fixed and stained with anti-myc antibody (A-C: sc-40, D: sc-789, Santa Cruz Biotechnology) and DAPI (shown in white in A and B to visualize faint ancillary kinetoplasts). A: PNT1::myc over-expressing cells exhibiting ancillary kinetoplasts (red arrowheads). Two of the three show PNT1 staining in both normal kinetoplasts (one as a doublet) and kinetoplast fragments. B: PNT1::myc/Mitotracker doubly stained cells. The bottom cell lacks a complete kinetoplast, retaining only a kinetoplast fragment near the posterior extremity (red arrowhead). C: A 1N1K cell showing nuclear PNT2 localization (N) and a 2N2K cell showing PNT2 localization to the spindle midzone (arrow). D: Co-staining of PNT2::myc with the KMX1 anti-tubulin antibody, using an alternative fixation protocol to visualize mitotic spindles (see Materials and Methods).

The proteins encoded by PUF9 target transcripts might be expected to function in similar processes since they are co-regulated in the cell cycle. To determine if this is the case, PNT1 and PNT2 were expressed as C-terminally myc-tagged proteins in PC cells. LIGKA is already known to be associated with the kDNA [17]. Remarkably, PNT1::myc was also found either forming a doublet closely flanking the kDNA or overlapping it (Figure 7A). We cannot rule out that the apparently overlapping signals are actually doublets orientated in-line with the kDNA as seen from above. Interestingly, when PNT1::myc was over-expressed, very small and faintly staining additional kinetoplasts appeared in 33% of cells after 8 hours, relative to 5% in uninduced cells. These were similar in size and localization to the “ancillary kinetoplasts” observed at low frequency in some other kinetoplastids [41]. They stained for PNT1::myc (red arrowheads, Figure 7A and 7B), and unlike normal kinetoplasts, were often mislocated anterior to the nucleus. These extra structures could represent degenerating kinetoplasts retained from a sister cell during division, fragmented kinetoplasts, or semi-synthesized kinetoplasts formed by aborted, late re-replication. A minor proportion of cells (∼1-3%, only marginally higher than in uninduced cells) lacked a normal kinetoplast, possessing only ancillary kinetoplasts or no kinetoplast at all. Total myc signal was dramatically increased in these cells and was localized throughout the mitochondrion (Figure 7B). Consistent with this, SignalP 2.0 HMM predicts a potential peptidase cleavage site 25 amino acids from the N-terminus, hinting at the presence of an N-terminal signal peptide.

PNT2::myc was expressed in PC cells using the same system described above. In clonal transfected cell lines, PNT2::myc appeared to localize to the mitotic spindle during mitosis and by the 2N2K stage it was seen in an elongated structure mid-way between the two nuclei (Figure 7C). Co-staining cells with the KMX1 antibody, which reveals mitotic spindles, confirmed that this structure was the mitotic spindle midzone (Figure 7D). The slightly granular appearance of cells here is an artifact of acetic acid fixation. No obvious over-expression phenotype was seen for PNT2::myc. However, PNT-2::myc was only expressed at low levels as seen by western blots of multiple transfected clones (not shown), which could preclude generation of an over-expression phenotype. We cannot rule out the presence of PNT2::myc in the cytoplasm since there was some diffuse cytoplasmic signal, but this was similar to the background signal seen in untransfected controls.

## Discussion

The increased multiplicity of protein function in eukaryotes has been proposed to be due partially to the replacement of inflexible prokaryotic polycistronic regulatory operons with gene-specific combinations of *cis*-acting regulatory elements [14]. The kinetoplastids may at first appear to challenge this paradigm of individualized gene regulation in eukaryotes, since they transcribe virtually all genes in large polycistrons that are even larger than operons of prokaryotic species. However, the kinetoplastid genomes are approximately two-fold enriched in genes encoding Pumilio domain and CCCH zinc finger proteins, relative to unicellular organisms that show transcriptional control, suggesting that these RNA-binding proteins have stepped in to replace transcription factors in regulating gene expression. This idea is supported by the current study where a small group of mRNAs that are likely to function in coordinated biological processes is shown to be under the control of a common upstream regulator, PUF9.

The temporal expression and localization of the proteins encoded by the PUF9 target transcripts indicates that they function in certain organelles (the nucleus, mitotic spindle and kinetoplast) at a specific time in the cell cycle (late S-phase/G2 phase). In kinetoplastid cells, the copy numbers of kinetoplasts and other major organelles and cellular structures are stringently maintained at one per G1 phase cell, and their replication is coordinated with each other and coupled to that of the cell. We hypothesize that PUF9 switches on the expression of target genes in late S-phase of the cell cycle to ensure simultaneous performance of their respective functions in organelle replication or division. Three lines of evidence support a role for PUF9 in co-coordinating cell-cycle governed replicative processes: firstly, the extra nuclei and kinetoplasts seen in BS cells when PUF9 is knocked down indicate that organelle replication is de-coupled from cell division. Secondly, PUF9 drives the upregulation of its target transcripts specifically in mid- to late- S-phase. Thirdly, bypassing PUF9 regulation by directly interfering with the expression of the downstream LIGKA or PNT1 proteins results in aberrant kinetoplast DNA content [17] or copy-number (shown here).

The parallels between two PUF9 targets, *LIGKA* (characterized previously [16],[17]) and *PNT1*, are particularly striking. Both encode proteins localized to the kinetoplast and both probably function in kinetoplast replication as indicated by their over-expression or knockdown phenotypes. The kinetoplast DNA is unique in nature in that it is a disc-shaped network of open circular DNA molecules, concatenated together with a “chain mail” topology (reviewed in [42]). It also has a unique mechanism of replication: individual minicircles disassociate from the network, migrate to the posterior kinetoflagellar zone where they are replicated, and then migrate back to one of the two “antipodal” sites which flank the disk at its perimeter, and where topoisomerase-mediated minicircle reattachment to the kinetoplast occurs. It has been hypothesized that LIGKA seals the final nick in replicated kDNA minicircles, re-licensing them for replication [16]. If so, this process would require some temporal and spatial regulation to prevent re-licensing in the vicinity of active minicircle replication machinery in the same cell cycle. Similarly, the fact that PNT1 over-expression results in an observable defect suggests that a tight reign on its protein levels must also be maintained to avoid negative consequences for the cell. We speculate that these shared requirements for tightly controlled expression may explain the involvement of PUF9 in regulating these two genes. Interestingly, the doublet that PNT1 sometimes forms, flanking the kinetoplast disc, is similar to that seen for proteins belonging to the antipodal sites where newly replicated minicircles are reattached to the kinetoplast.

PNT2::myc displayed an interesting cell-cycle dependent dynamic localization, being present in the nucleus in pre-mitotic cells but localized to the spindle midzone in post-mitotic cells. Because its mRNA levels peak at around mid- to late- S-phase, the protein levels of endogenous PNT2 might be expected to peak shortly afterwards, probably co-inciding with this relocalization during mitosis. Similar localization patterns have been reported for certain other proteins such as TbNOP86, a nucleolar protein that localizes to the spindle during cytokinesis and whose RNAi phenotype resembles that of PUF9, *i.e*. an increase in G2 phase and polyploid cells [43]. Some chromosomal passenger proteins such as TbCPC1 and TbCPC2 also display similar localization during mitosis, although they additionally relocalize to a dot on the cleavage furrow during cytokinesis, which we could not detect for PNT2::myc. These proteins are involved in spindle function and cytokinesis as their suppression in PCs leads to mitotic spindle abnormalities and accumulation of G2-phase cells [44]. Given its similar localization and temporal expression, we speculate that PNT2 may have a similar function related to the timing of mitotic spindle assembly or disassembly.

In addition to being regulated at the mRNA level, LIGKA, PNT1 and PNT2 are probably regulated at the protein level. LIGKA protein is known to be relatively unstable [16], while levels of tagged PNT1 in the PNT1::myc over-expressing cells seem to be self-limiting, in light of the fact that many cells displaying the associated phenotype no longer expressed detectable protein at the time of fixation (*e.g.* the top cell in Figure 7A, which possesses an anterior kinetoplast fragment but does not express PNT1::myc). Further, the massive increase in PNT1::myc protein levels in the mitochondrion of cells lacking a normal kinetoplast may mean that the kinetoplast not only sequesters PNT1 but also suppresses PNT1 expression at the protein level, although here we have only demonstrated an associative, rather than causal, relationship between kinetoplast loss and increased PNT1 protein. Additionally, the ancillary kinetoplasts we observed, while capable of sequestering PNT1, were not by themselves associated with repressed PNT1 protein levels. In general, rapid protein degradation for cyclically regulated proteins should allow a sharper peak in protein levels to occur at the proper time.

Puf proteins in other eukaryotes generally act to destabilize their target transcripts. However, PUF9 was shown to stabilize its target transcripts, and similarly, to stabilize a reporter transcript carrying the 3′ UTR of *PNT1*. The presence of the UUGUACC motif (common to all PUF9 target 3′ UTRs) is essential for regulation by PUF9. The fact that mutations in this motif appear to stabilize the transcript, rather than destabilizing it, suggests that the motif recruits a destabilizing factor that is then inhibited by PUF9. Probable candidates for this destabilizing factor include the other Puf-domain proteins, since as mentioned, they generally act to destabilize targets, and are quite likely to bind the same motif as PUF9 because Puf proteins usually bind a conserved “UGUA”-containing motif [39]. This would potentiate a simple binding-site competition model of regulation whereby PUF9 and the destabilizing factor compete for binding at the same RNA motif. Other more complex mechanisms are also plausible, and it is worth noting that the 3′ UTR of PNT-1, while capable of conferring cell-cycle regulation onto a reporter transcript, did not confer exactly the same expression as the native transcript, which implicates the 5′ UTR and ORF, and perhaps even pre-mRNA sequences, in fine-tuning post-transcriptional regulation of *PNT1*.

The means by which PUF9 seems to be most active in late S-phase is of particular interest if PUF9 is to be placed within a cell-cycle regulatory network. Higher expression of PUF9 protein during S-phase may occur, and indeed there was an indication that *PUF9* transcript levels oscillate with the cell cycle. However, the magnitude of *PUF9* transcript upregulation in S-phase seemed to be insufficient to fully explain that of its downstream targets. While regulation may occur at the translational level, a suitable antibody against PUF9 could not be raised to test this. Post-translational modifications such as phosphorylation, ubiquitination *etc.* may also play a role, although a V5-tagged PUF9 protein showed no changes in electrophoretic mobility (which might indicate post-translational modification) over the cell cycle, and a recent analysis of the *T. brucei* phosphoproteome did not identify PUF9 among the set of phosphorylated proteins [45]. However, all these possible modes of regulation might not act only on PUF9 but rather on factors cooperating with PUF9 in regulating transcriptional stability, for instance the factor hypothesized above to act contrary to PUF9 to destabilize the target transcripts.

The characterization of PUF9 target transcripts in *T. brucei* appears to be revealing a post-transcriptional regulon of genes involved in coordinating the replication of subcellular structures in the cell cycle. This is regulated independently to the set of cell-cycle-regulated transcripts investigated by several other groups [10],[11],[12],[13] containing *DHFR*, *LIGKB*, *TOP2* and *RPA1*, because the timing of peak expression of the PUF9 targets is significantly later in the cell cycle (as was first observed when the expression of *LIGKB* was compared to that of *LIGKA* in *C. fasciculata* [16]). The three PUF9 targets encode kinetoplast- or spindle- associated proteins, are co-regulated in the cell cycle, and perturbations in expression of at least two of them (*PNT1* and *LIGKA* [17]) cause defects in kinetoplast replication. The main function of PUF9 is probably to ensure simultaneous function of its target genes during late S-phase in order to temporally coordinate certain processes in organellar and cellular replication. In particular, the putative roles for PUF9 target proteins in kinetoplast replication and the mitotic spindle implicates PUF9 in synchronizing kinetoplast maturation and mitotic spindle function. The further investigation of the upstream regulatory network and downstream effectors will lead to insights into how trypanosomes coordinate the replication of their organelles with that of the entire cell, and how they regulate gene expression in general without transcriptional control.

## Supporting Information

Figure S1 Effect of *PUF9* over-expression or knockdown on target transcripts in BS cells. *PUF9* over-expressing (*PUF9*↑) or *PUF9* RNAi (*PUF9*↓) BS cells were uninduced or induced with tet for 24 hours prior to RNA isolation and Northern blotting. Results from a representative experiment are shown. The blot was probed for *PUF9* and PUF9 target genes. A replicate blot was probed separately for some transcripts (*).
(0.31 MB TIF)

Figure S2 Effect of *PUF9* RNAi on target transcripts in PC cells. A: PC cells inducibly targeting *PUF9* by RNAi were cultured for 24 hours with or without tet induction, prior to RNA isolation and Northern blotting. Duplicate blots were probed for *PUF9* and PUF9 target transcripts as well as the *SRP* RNA as a loading control. One allele of *PUF9* in the parent cell line was *in situ*-tagged with the V5 epitope (to facilitate checking clones for RNAi), possibly explaining the two slightly different sized *PUF9* transcripts. B: Down-regulation of mRNA in *PUF9* RNAi PCs is restricted to PUF9 target transcripts. Total RNA was isolated from two *PUF9* RNAi PC clones, with or without induction of RNAi by 24 hours of tet treatment. Levels of the PNT1 mRNA, the *SRP* RNA, and a control mRNA (the abundant *Tb927.5.2560* transcript), were compared by Northern blotting and hybridization.
(5.19 MB TIF)

Figure S3 Effect of *PUF9* RNAi on abundance of CAT reporter protein expressed from a *PNT1* 3′ UTR - bearing transcript. A: BS cells, expressing the *CAT::PNT1-3′ UTR* reporter transcript, were induced with tet to suppress *PUF9* by RNAi. Approximately 2×10^6^ cells were collected after 0, 24 or 48 hours of RNAi induction by tet and analyzed by SDS-PAGE and western blotting. CAT was visualized by probing with rabbit anti-CAT antibody (5 Prime 3 Prime; 1:2000 dilution), or rabbit anti-*T. brucei* aldolase antisera (1:50000 dilution) as a loading control. A HRP-conjugated anti-rabbit IgG antibody was used to detect the primary antibody in conjunction with the ECL detection system (GE Healthcare). B: BS cells were again induced with tet to suppress *PUF9* by RNAi, and samples were taken immediately or after 24 hours of RNAi for RNA isolation and Northern blotting. The blot was probed for the *CAT* transcript and the *SRP* RNA as a loading control.
(0.41 MB TIF)
