# Supplementary figures and images for: Trypanosoma brucei PUF9 Regulates mRNAs for Proteins Involved in Replicative Processes over the Cell Cycle
Source: PLoS Pathog. 2009 Aug 28;5(8):e1000565. doi: 10.1371/journal.ppat.1000565 (PMC2727004; doi:10.1371/journal.ppat.1000565)

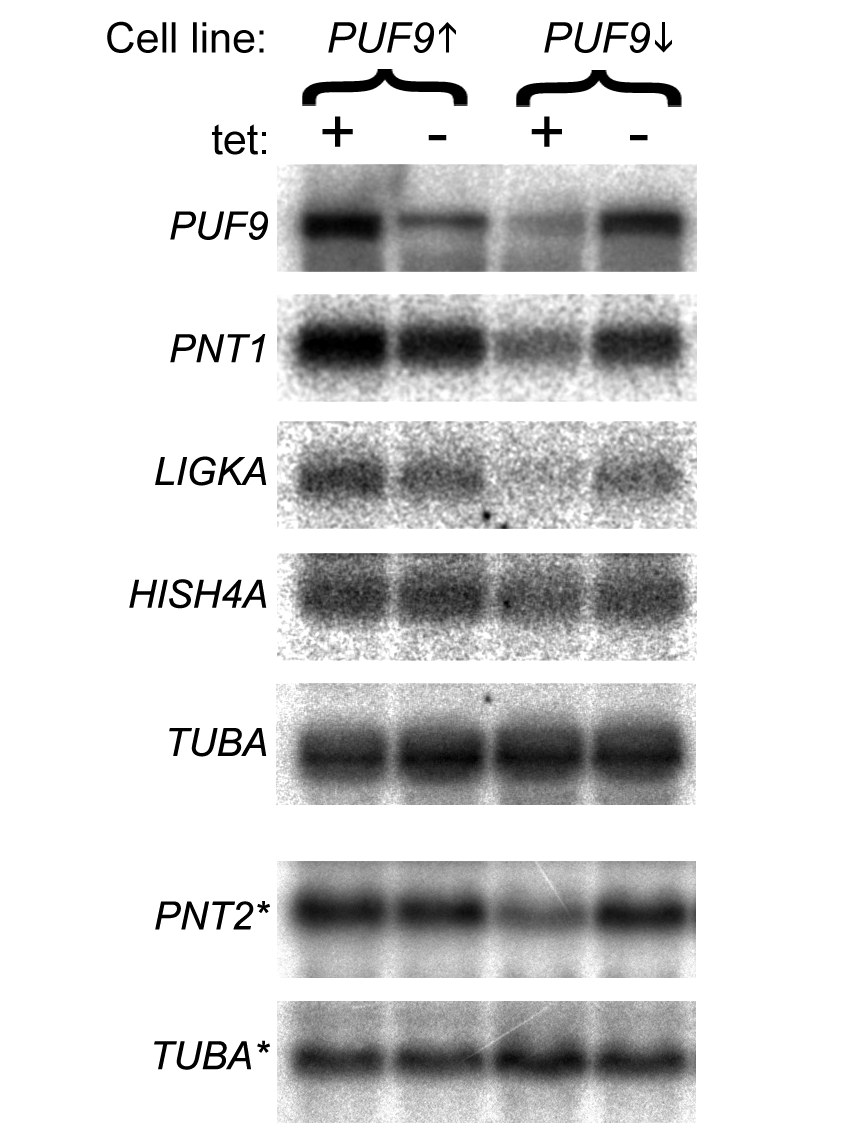

Supplement: Figure S1 — Effect of PUF9 over-expression or knockdown on target transcripts in BS cells. PUF9 over-expressing (PUF9↑) or PUF9 RNAi (PUF9↓) BS cells were uninduced or induced with tet for 24 hours prior to RNA isolation and Northern blotting. Results from a representative experiment are shown. The blot was probed for PUF9 and PUF9 target genes. A replicate blot was probed separately for some transcripts (*). (0.31 MB TIF) [file ppat.1000565.s001.tif]

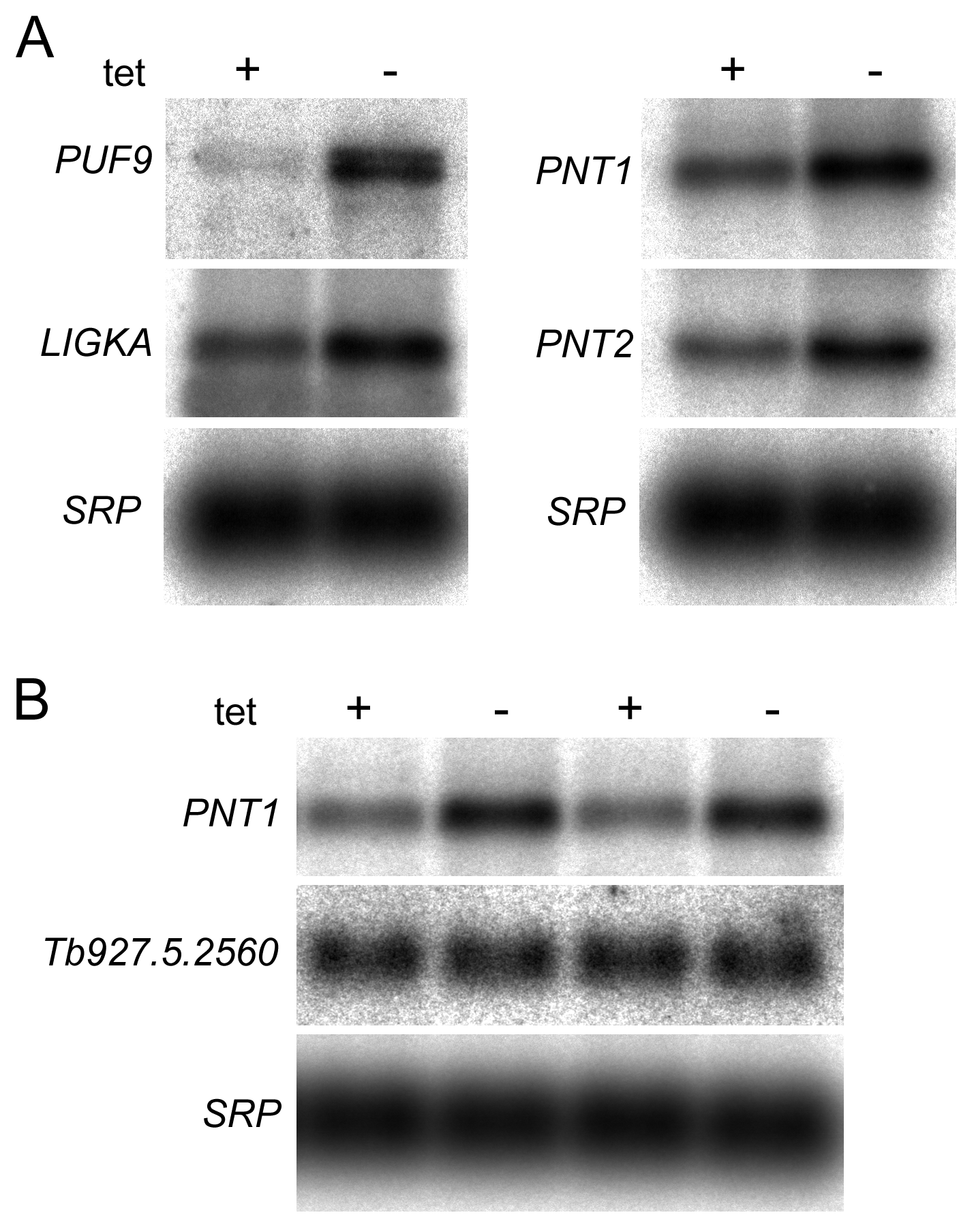

Supplement: Figure S2 — Effect of PUF9 RNAi on target transcripts in PC cells. A: PC cells inducibly targeting PUF9 by RNAi were cultured for 24 hours with or without tet induction, prior to RNA isolation and Northern blotting. Duplicate blots were probed for PUF9 and PUF9 target transcripts as well as the SRP RNA as a loading control. One allele of PUF9 in the parent cell line was in situ-tagged with the V5 epitope (to facilitate checking clones for RNAi), possibly explaining the two slightly different sized PUF9 transcripts. B: Down-regulation of mRNA in PUF9 RNAi PCs is restricted to PUF9 target transcripts. Total RNA was isolated from two PUF9 RNAi PC clones, with or without induction of RNAi by 24 hours of tet treatment. Levels of the PNT1 mRNA, the SRP RNA, and a control mRNA (the abundant Tb927.5.2560 transcript), were compared by Northern blotting and hybridization. (5.19 MB TIF) [file ppat.1000565.s002.tif]

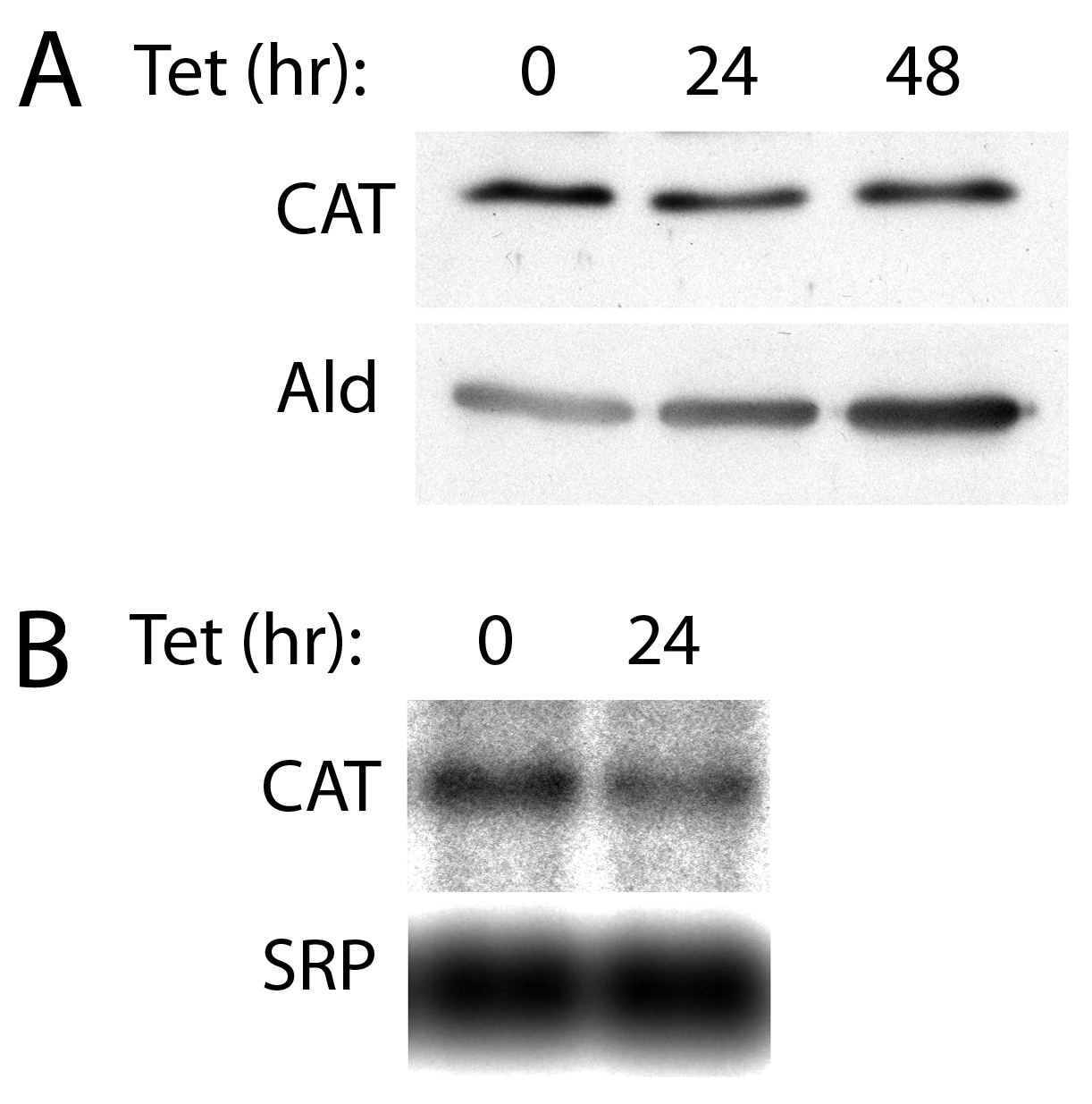

Supplement: Figure S3 — Effect of PUF9 RNAi on abundance of CAT reporter protein expressed from a PNT1 3′ UTR - bearing transcript. A: BS cells, expressing the CAT::PNT1-3′ UTR reporter transcript, were induced with tet to suppress PUF9 by RNAi. Approximately 2×10^6^ cells were collected after 0, 24 or 48 hours of RNAi induction by tet and analyzed by SDS-PAGE and western blotting. CAT was visualized by probing with rabbit anti-CAT antibody (5 Prime 3 Prime; 1:2000 dilution), or rabbit anti-T. brucei aldolase antisera (1:50000 dilution) as a loading control. A HRP-conjugated anti-rabbit IgG antibody was used to detect the primary antibody in conjunction with the ECL detection system (GE Healthcare). B: BS cells were again induced with tet to suppress PUF9 by RNAi, and samples were taken immediately or after 24 hours of RNAi for RNA isolation and Northern blotting. The blot was probed for the CAT transcript and the SRP RNA as a loading control. (0.41 MB TIF) [file ppat.1000565.s003.tif]
